# Propensities and Second Order Uncertainty: A Modified Taxi Cab Problem

**DOI:** 10.3389/fpsyg.2020.503233

**Published:** 2020-10-20

**Authors:** Stephen H. Dewitt, Norman E. Fenton, Alice Liefgreen, David A. Lagnado

**Affiliations:** ^1^Department of Experimental Psychology, University College London, London, United Kingdom; ^2^School of Electronic Engineering and Computer Science, Queen Mary University of London, London, United Kingdom

**Keywords:** causal Bayesian networks, second order uncertainty, propensity, uncertainty, confirmation bias

## Abstract

The study of people’s ability to engage in causal probabilistic reasoning has typically used fixed-point estimates for key figures. For example, in the classic taxi-cab problem, where a witness provides evidence on which of two cab companies (the more common ‘green’/less common ‘blue’) were responsible for a hit and run incident, solvers are told the witness’s ability to judge cab color is 80%. In reality, there is likely to be some uncertainty around this estimate (perhaps we tested the witness and they were correct 4/5 times), known as second-order uncertainty, producing a distribution rather than a fixed probability. While generally more closely matching real world reasoning, a further important ramification of this is that our best estimate of the witness’ accuracy can and should change when the witness makes the claim that the cab was blue. We present a Bayesian Network model of this problem, and show that, while the witness’s report does increase our probability of the cab being blue, it simultaneously decreases our estimate of their future accuracy (because blue cabs are less common). We presented this version of the problem to 131 participants, requiring them to update their estimates of both the probability the cab involved was blue, as well as the witness’s accuracy, after they claim it was blue. We also required participants to explain their reasoning process and provided follow up questions to probe various aspects of their reasoning. While some participants responded normatively, the majority self-reported ‘assuming’ one of the probabilities was a certainty. Around a quarter assumed the cab was green, and thus the witness was wrong, decreasing their estimate of their accuracy. Another quarter assumed the witness was correct and actually increased their estimate of their accuracy, showing a circular logic similar to that seen in the confirmation bias/belief polarization literature. Around half of participants refused to make any change, with convergent evidence suggesting that these participants do not see the relevance of the witness’s report to their accuracy before we know for certain whether they are correct or incorrect.

## Introduction

While causal Bayesian reasoning, and reasoning under uncertainty in general are major research programs within the judgment and decision-making literature, problems presented to participants have typically only studied this under first order uncertainty (also known as ‘risk’ in the economics literature). For example, the participant might be given a betting choice between a sure win of £25 or a 33% chance of £100 (e.g., Kahneman and Tversky, 1979). Here, while in the latter option it is uncertain whether we will get the £100, we can quantify this uncertainty precisely, and the problem thus yields simply to an expected utility calculation. But what if we did not know for certain what the chance of getting the £100 was? For example, suppose the probability was based on the outcome of some exotic asymmetrical die. Suppose also that we don’t understand the mechanics of the die, but we have observed 3 rolls, with only 1 leading to a win. While 33% might still be our best guess, with such a small sample size to estimate this, a substantial range of other probabilities are possible. How would this affect our decision over which bet to take? This uncertainty about our first order uncertainty is known as second order uncertainty (e.g., Kleiter, 2018), and we currently know little about how classic findings in the judgment and decision-making literature apply under such conditions.

Kahneman and Varey (1990) divided uncertainty along another dimension: internal uncertainty and external uncertainty (see also Juanchich et al., 2017). While internal uncertainty comes from our own ignorance about the world (e.g., the mechanics of the above die), external uncertainty comes from the propensity for an external causal system (such as the exotic die) to produce various outcomes or effects (e.g., a ‘win’). However, much we reduce our internal uncertainty about the mechanics of the die, we will only ever be able to predict what face will land up according to those propensities and never be able to guarantee a given outcome. This example illustrates an interaction between these two types of uncertainty which was not discussed in that paper. In this situation we have internal uncertainty about the propensity (external uncertainty) of the die to produce a ‘win.’ This is an extremely common situation – in fact, outside of contrived situations such as (standard) die rolls and coin flips, our estimates of the propensities for external causal systems to produce a given effect often comes with some internal (second order) uncertainty. Consider the propensity for a prisoner to reoffend or a patient to relapse or suffer complications. In each case the individual presumably has some true propensity (although this may fluctuate in a complex manner over time and context) but we only have limited information from which to estimate it. We are principally interested here in individuals’ ability to update propensity estimates in light of new information, i.e., update first order uncertainty estimates under conditions of second order uncertainty.

Approaches used to solve first order probability problems typically cannot be applied to second order problems. Knight (1921) gave the example of a picnic as a situation where first order techniques (e.g., expected utility calculations) were not workable. However, a true investment scenario, as opposed to the example we began the paper with is also insightful. When deciding whether to invest, one may use current and historical stock market figures, one’s feelings about and trust in the CEO and other bits of information such as a tip from an insider and other known markers of health. Under such conditions the probability of a positive return on investment cannot be reduced to a first order point estimate with no variance. Indeed, Mousavi and Gigerenzer (2014, 2017) have lamented the fact that while the vast majority of the experimental economics literature has aimed to study a higher order uncertainty problem (reasoning about business and economics), it has used experimental materials featuring only first order uncertainty. If we want to understand real world human reasoning outside of casino gambling, we must incorporate higher order uncertainty into the problems we use to study this.

Similarly, while second order uncertainty has been written about in the context of causal Bayesian reasoning within the judgment and decision-making literature (e.g., Gigerenzer and Hoffrage, 1995; Welsh and Navarro, 2012; Kleiter, 2018), reasoning under these conditions has rarely been studied, and experiments aiming to study real world reasoning have also typically done this using problems with only first order uncertainty. For example, in the classic taxi cab problem (Tversky and Kahneman, 1974; Bar-Hillel, 1980), solvers are asked to reason about whether a cab involved in a hit and run accident was from the ‘blue’ company (as opposed to the ‘green’) in light of a population base rate (which suggests green cabs are more common) and an eye witness report (which claims a blue cab was involved). Solvers are told that the witness was tested for their ability to judge cab color, and that their ability was found to be 80%. A version of this can be seen below:

A cab was involved in a hit-and-run accident at night. Two cab companies, the Green and the Blue, operate in the city.

You are given the following data: 90% of the cabs in the city are Green and 10% are Blue.

A witness identified the cab as Blue. The court tested the reliability of the witness under the circumstances that existed on the night of the accident and concluded that the witness correctly identified each of the two colors 80% of the time and failed 20% of the time.

What is the probability that the cab involved in the accident was Blue rather than Green?

In order to solve the problem, participants have to integrate the figure regarding the proportion of blue cabs in the city (not many: 10%) with the contradictory evidence of the witness’s claim that the cab was blue and their accuracy (quite good: 80%) to arrive at a final probability that the cab involved in the incident was blue. A major finding of the original paper was that many participants neglected the population base rate data entirely in their final estimate, simply giving the witness’s accuracy (80%) as their answer. Subsequent work has found that base rates more specific to the incident [e.g., in the area of the incident rather than in the city as a whole (Bar-Hillel, 1980)] and more causally related [e.g., where green cab drivers are known to get into more accidents, rather than just being more prevalent (e.g., Ajzen, 1977)] reduce base rate neglect.

In the similar medical diagnosis problem (e.g., Casscells et al., 1978; Gigerenzer and Hoffrage, 1995), solvers are asked to reason about whether a patient has cancer, given a population base rate (suggesting cancer is unlikely) combined with a positive test result. The solver is told that the false positive error rate of the mammogram test is 5%. In reality of course, there is likely to be some uncertainty around the probability estimates of both the witness’s accuracy in the taxi cab problem and the false positive rate of the medical test. Our estimates therefore should look more like a distribution than a single point. While 80%/5% might provide the mean, or our best guess, there will also be some variance around this, due to our ignorance (internal uncertainty). The degree of variance depends upon the quality and amount of information we have available. These two examples prove useful in demonstrating this. While it may seem plausible that the mammogram machine has been tested a great many times, perhaps thousands of times, and thus, variance in our estimate might be very small, this seems less plausible for the single witness in the taxi cab problem, where time and resources would heavily limit the number of tests possible. Furthermore, it seems unlikely that the exact circumstances of the crash could be replicated for testing purposes, further increasing our uncertainty in the estimate. While we may therefore be justified in approximating the 5% false positive rate as a fixed-point estimate with no variance to simplify the problem, this is unlikely to be reasonable for the taxi-cab problem.

**FIGURE 1:**
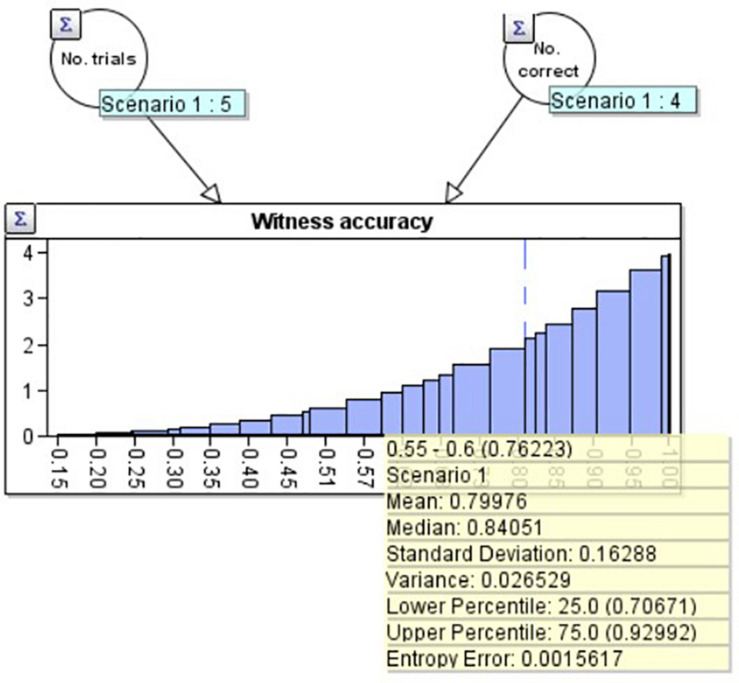
A beta distribution with mean 80.0% and standard deviation 16.3% created using the Agenarisk software.

For example, suppose the witness has been tested 5 times, getting 4 correct. This produces a distribution with a classical statistical mean of 80% and a standard deviation of 16.3%. We created such a distribution in the ‘AgenaRisk’ Bayesian network program, which can be seen in Figure 1. We use a beta distribution (Kleiter, 2018) based upon the two nodes above it: No. trials (5) on the left, and No. correct (4) on the right. The mean and other statistics associated with the distribution can be seen in the yellow summary box.

**FIGURE 2:**
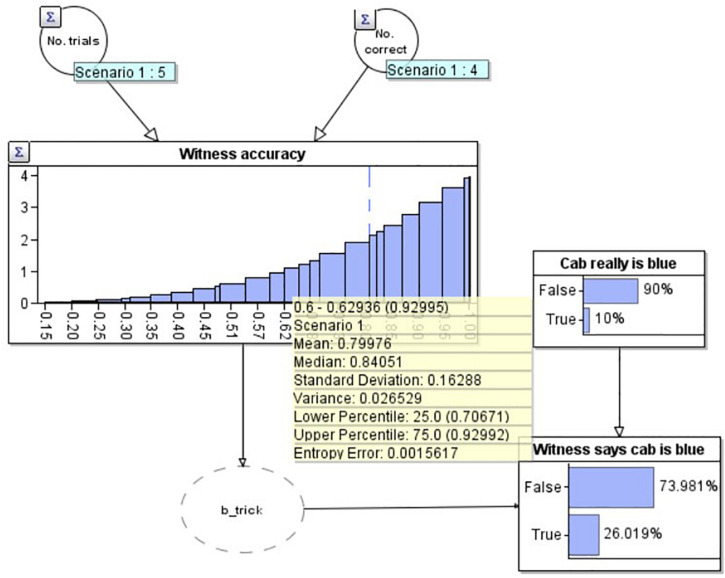
A Bayesian network depicting the modified taxi cab problem prior to the witness reporting the cab is blue.

Now that we know the initial distribution of our estimate of the witness’s accuracy, in order to model the full problem, we need to be able to update this distribution depending on whether the witness gets future reports correct or incorrect. We model this by expanding Figure 1 into a larger Bayesian network (BN: Figure 2). A BN is a directed graph whose nodes represent uncertain variables, and where an arc (or arrow) between two nodes depicts a causal or influential relationship [see Fenton and Neil (2018) for full details of BN’s]. In addition to the graph structure, each node has an associated probability table which defines the prior probability distribution for the associated variable, conditioned (where a node has parents) on its parent variables. When the state of a node is observed (e.g., the witness reports that the cab is blue) the known value is entered into the BN via an ‘observation’ and a propagation algorithm updates the probability distributions for all unobserved nodes. The ‘Bayesian’ in BN’s is due to the use of Bayes’ theorem in the underlying propagation algorithm.

In this diagram, our estimate for the witness’s accuracy has been connected to a node (‘Witness says cab is blue’) depicting whether the witness reports that the current cab is blue. The ‘b_trick’ node is simply a pragmatic software requirement to convert the witness’s accuracy distribution into a binary variable. The probability that the witness says the cab is blue is causally dependent upon both their accuracy, and the base rate, depicted in the above node ‘Cab really is blue.’ The current diagram depicts the situation before the witness makes their report. The best estimate that the cab is really blue at this point is just the base rate, 10%. Combining this and the witness’s accuracy, the model predicts a 74.0% chance that the witness will report that the cab is green.

**FIGURE 3:**
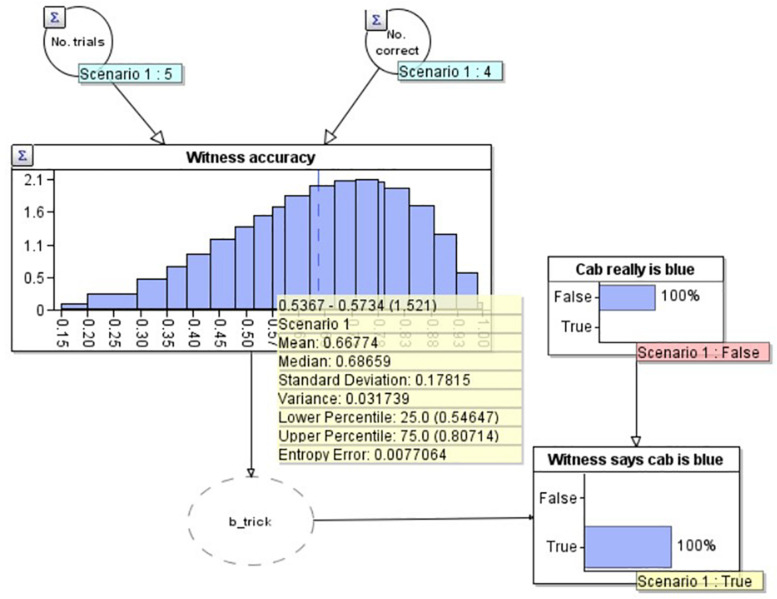
A Bayesian network model depicting the situation where the witness is incorrect about the cab being blue.

**FIGURE 4:**
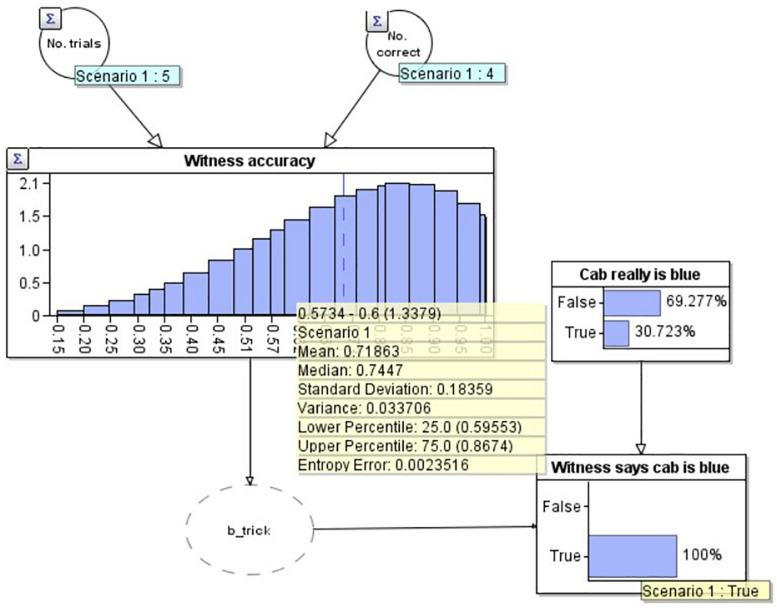
A Bayesian network model depicting the situation where the witness has reported the cab is blue but we are uncertain if they are correct or incorrect.

To demonstrate the workings of the model, in Figure 3 we add two observations to the model. Firstly, in the lower right, we set an ‘observation’ on the ‘Witness says cab is blue’ node that the witness has said the cab is blue (note that this now has a yellow label saying ‘True’). We have also set an observation on the ‘Cab really is blue’ node to make this ‘False’ i.e., as if we knew the cab really was green (and therefore the witness was incorrect). As would be expected in this scenario, our estimate of the witness’s accuracy goes down to 66.7% (see yellow summary box mean), as we would expect given that they now have 4 correct out of 6 (4/6 = 0.666….). Similarly, if the witness reports that the cab is blue, and we model that the cab really was blue (i.e., the witness is correct) by setting ‘Cab really is blue’ to ‘True’ (not depicted), the model provides an estimate of the witness’s accuracy of 83.3%, equivalent to getting 5 out of 6 correct (5/6 = 0.833). Of present interest however, is how the witness’s accuracy should change outside of these ‘certain’ bounds: when the witness reports that the cab is blue, but we don’t know for certain if they are correct or not (Figure 4).

In Figure 4 we have modeled the problem to include the witness’s report that the cab was blue, but without knowing the truth for certain (no ‘observation’ on the ‘Cab really is blue’ node). Not only, as expected, has the probability that the cab is blue increased (to 30.7% from 10.0%), but simultaneously, our estimate of the witness’s accuracy has reduced, to 71.9%, below the initial estimate (80.0%, i.e., 4/5) but not as low as the estimate if we knew for certain the witness was incorrect (66.7%, i.e., 4/6). The reason for this reduction is that the witness has made a claim which goes against the only other evidence we have (the base rate, which suggests that the cab is green with considerable strength). If the witness had instead claimed that the cab was green, our estimate of their accuracy would increase (to 82.9%), again less than if we knew for certain they were correct (83.3%).

This addition of second order uncertainty therefore gives the problem a more dynamic character than the original problem. Furthermore, it cannot be solved with a simple application of Bayes’ theorem, unlike the original taxi cab problem. It also has a potentially unintuitive dynamic: while we have enough trust in the witness to ‘use’ the information they provide as evidence that the cab was blue, we simultaneously reduce our trust in the witness’s ability to make this very judgment in future. To keep things initially simple, as can be seen, we do not model the prior for the cab being blue as having second-order uncertainty. As will be discussed later, the version of the problem we use justifies a fixed estimate for this (we have complete knowledge), however, versions with second order uncertainty here may also be interesting.

Our primary aim is to examine participant responses to this novel problem and their ability to reason about causal relationships under second order uncertainty, and particularly through that unintuitive dynamic which is typical of such problems. Lacking the assistance of software like the above, the precise normative answer will not be achievable by our participants. For this reason, and because we believe such numerical precision is unlikely to characterize real world reasoning, we are not interested in participants’ ability to do the mathematics, or the magnitude of their adjustments when they find out the witness reports the cab was blue. Instead we are interested only in the direction of their adjustments for the two main estimates (the witness’ true accuracy level and the probability the cab is blue) and particularly whether they recognize that the witness’s accuracy should be reduced. We will also request participants to explain their reason for their responses and provide several follow up questions to probe their representation and processing of the problem, in line with recent calls for more process-oriented work within this literature (Johnson and Tubau, 2015; McNair, 2015).

## Materials and Methods

### Participants

One hundred and thirty-one participants (43.5% female), recruited from Prolific Academic (paid £9 per hour), took part in the study, with an average age of 27.8 (*SD* = 9.8). No participants were removed from the statistical analyses.

### Design

All 131 participants saw the same version of the study. Participants were sub-divided in the analysis based upon their response to the key question of interest, and we used a range of numerical, and open and closed qualitative data to uncover the cognitive processes behind these different response types.

### Materials and Procedure

All materials and data can be found in a public repository at https://osf.io/q68cu/. Participants were firstly presented with the information sheet and once clicking ‘Next’ to indicate their consent, were presented with the hit and run scenario. Participants were only able to move forward in the experiment, and could not go back and check previous pages. They were first told that a CCTV camera had made a ‘partial read’ on a taxi cab’s license plate fleeing the scene, and that only 10 cabs matched: 1 belonging to the blue company, 9 to the green company (giving a first order probability with no second order uncertainty, assuming it is trusted). They were then asked to give a percentage estimate using a slider that the cab was blue based only on this information. On a new page, they were then told a witness had come forward, and were given information on the witness’s accuracy. Participants were told the police had tested the witness five times, and the witness was correct four times. They were then asked to estimate the witness’s true accuracy from this. To encourage participants not to see the initial accuracy value as fixed (i.e., overly subscribe to the law of small numbers), we included the following text to emphasize that we have only a limited estimate of their true accuracy.

However, we only have 5 trials to estimate this. It’s possible they got lucky once or twice during the test. If we ran 100 trials we would have a more reliable estimate. Perhaps they would get 70 correct, or even 90.

Participants were then asked to use two sliders to give an estimate of the witness’s ‘true’ accuracy (0–100%), and separately, provide a (0–100%) confidence that that estimate “would be the witness’s true accuracy level if we ran a lot more trials.” Only after providing these two prior estimates, participants were told on a new page that the witness had claimed the cab was blue.

**FIGURE 5:**
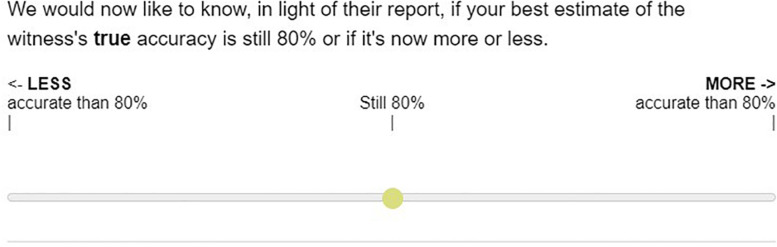
Image of the slider mechanism used for participants to adjust their estimate for the witness.

Following this, participants were first asked to update their estimate that the cab was blue and then on a separate page, update their estimate of the witness’s accuracy. In both cases key information was re-summarized. Instead of being asked to give a numerical value at this point, participants indicated on a sliding scale (Figure 5) whether they wanted to stick with their original value or increase their estimate. Participants were forced to answer all questions in the survey and were not able to proceed with the experiment if they simply left the slider in place. If they wished to make no change and keep their original estimate they first had to move the slider to activate it, then move it back to the center.

The degree to which participants moved the slider was not of importance, and was only included to allow participants to express themselves and to reduce the chance of participants who wanted to make a very small change choosing to make no change. This approach was used to discourage participants from attempting a mathematical treatment of the problem, which we strongly believe cannot be the way people solve real life problems of this type. Instead, we wanted to capture intuitive feelings of whether the two variables both go up, both go down, stay the same, or (as predicted by the normative model) the probability of the cab being blue goes up, while the accuracy of the witness goes down. It is at this coarser level at which participants responses were judged. For both estimates, on the same page, participants were asked to explain their reasoning in an open text box.

After making posterior estimates, participants were asked in a multiple-choice format whether, when reasoning through the problem they had (A) Assumed the cab was green, (B) Assumed the witness was correct or (C) Neither/Other. The order of these options was randomized.

Participants were finally told on a separate page that after the investigation had concluded it turned out the cab really was green and so we now know the witness was incorrect this time. Participants were then asked again whether they wished to adjust the witness’s accuracy using the same slider and were again provided with an open text box to explain their reasoning.

## Results

### Manipulation Checks/Priors

**FIGURE 6:**
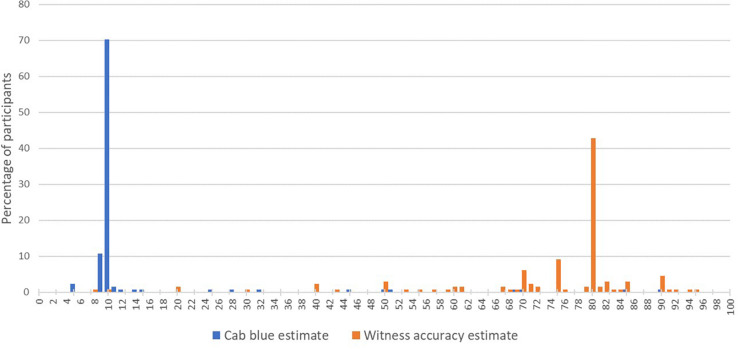
A histogram representing participant prior estimates of the probability the cab is blue (blue) and witness accuracy (orange).

After being provided with the prior for the cab being blue, participants were asked to indicate on a sliding scale the probability the cab was blue and 82.4% chose 9, 10, or 11%, suggesting a high level of ‘acceptance’ of the prior figure (Mean = 15.6%, *SD* = 16.2%). Participants were also asked to do the same for the witness’s accuracy, after being given the figures on the court’s testing of them and 45.8% chose 79, 80, or 81% (Mean = 73.5%, *SD* = 15.5%). The distribution of responses for both can be seen in Figure 6. Participants were also asked to express their confidence that this figure represented the witness’s true accuracy, which produced a mean of 68.6% (*SD* = 20.7%).

### Posteriors

Once the witness reports that the cab was blue, participants were firstly asked to adjust their estimate that the cab was blue, and then the witness’s accuracy. Out of all participants, 64.9% increased the probability that the cab was blue. Our primary interest, however, was what change they made to their estimate of the witness’s accuracy. Only 21.4% reduced this, while 55.7% made no change, and 22.9% increased it. In the following we analyze these three sub-groups according to their responses to a range of questions to attempt to understand their cognitive processes. Figures to support these analyses can be seen in Table 1 and will be referred to throughout.

**TABLE 1:** Participant responses to a range of questions sub-divided by their initial response to altering the witness’s accuracy.

	Reduce	No change	Increase
Total *N*	28 (21.4%)	73 (55.7%)	30 (22.9%)
	**Cab/witness statistics provided**		
*(Mean)* Cab blue estimate	9.5% (0.3%)	16.8% (2.0%)	18.2% (3.6%)
*(Mean)* Witness accuracy estimate	76.0% (1.9%)	73.3% (2.1%)	71.6% (2.6%)
*(Mean)* Confidence	67.1% (2.9%)	68.8% (2.7%)	69.9% (3.3%)
	**Witness reports cab is blue**		
*(Proportion)* Increasing estimate cab blue	67.9% (9.0%)	56.2% (5.8%)	83.3% (6.9%)
*(Proportion self-reported assuming):*			
Witness correct	25.0% (8.3%)	60.3% (5.8%)	80.0% (7.4%)
Cab green	46.4% (9.6%)	15.1% (4.2%)	3.3% (3.3%)
Neither/other	28.6% (8.7%)	24.7% (5.1%)	16.7% (6.9%)
	**Told cab actually green**		
*(Proportion)* Reducing witness accuracy	78.6% (7.9%)	52.1% (5.9%)	73.3% (8.2%)

*In brackets on the left it is indicated whether the figure provides the mean response, or the proportion providing a particular response (in the case of non-continuous outcomes). Questions are in chronological order and the delivery of key pieces of information is indicated. Standard errors for estimates are included in brackets.*

#### Statistical Comparisons

We proceed in the following from top to bottom. In the second row of Table 1 [(Mean) Cab blue %] we can see the average estimate that the cab was blue made by each of the three response types before the witness’s report. A univariate analysis was run to test the effect of ‘Response type’ on ‘(Mean) Cab blue %’ [*F*(2,128) = 2.6, *p* = 0.08]. Pairwise comparisons compared ‘No Change’ to ‘Reduce’ (*p* = 0.04) and ‘No Change’ to ‘Increase’ (*p* = 0.70) and ‘Reduce’ to ‘Increase’ (*p* = 0.04). Univariate analyses were also run to test the effect of ‘Response type’ on the third row, their estimate of the witness’s accuracy before the witness’s report [(Mean) Witness accuracy] [*F*(2,128) = 0.6, *p* = 0.55] and separately on row four, their confidence that this estimate was equal to the witness’s true accuracy, [(Mean) Witness confidence] [*F*(2,128) = 0.13, *p* = 0.88].

Moving to row five, only a single individual reduced their estimate of the cab being blue after the witness’ report. The remainder either made no change, or increased their estimate. A binary logistic regression was run to test the effect of ‘Response type’ on the proportion of individuals increasing their estimate of the cab being blue [Wald *X*^2^ (2) = 6.6, *p* = 0.04]. Pairwise comparisons were then run to compare ‘No Change’ and ‘Increase’ [Wald *X*^2^ (1) = 6.3, *p* = 0.01], ‘No Change’ and ‘Reduce’ [Wald *X*^2^ (1) = 1.1, *p* = 0.29], and ‘Reduce’ and ‘Increase’ [Wald *X*^2^ (1) = 1.8, *p* = 0.18].

We now move to rows six, seven, and eight, representing the proportion of each response type who chose either ‘Assumed the witness was correct,’ ‘Assumed the cab was green,’ or ‘Neither/other’ when faced with this question. Binary logistic regressions were run to test the effect of ‘Response type’ on assuming the witness was correct [Wald *X*^2^ (2) = 16.2, *p* < 0.001], on assuming the cab was green [Wald *X*^2^ (2) = 14.9, *p* < 0.001] and on ‘Neither/other’ [Wald *X*^2^ (2) = 1.2, *p* = 0.55]. Examining the assumption that the witness was correct, pairwise comparisons were run to compare ‘No Change’ to ‘Increase’ [Wald *X*^2^ (1) = 3.5, *p* = 0.06], ‘No Change’ to ‘Reduce’ [Wald *X*^2^ (1) = 9.3, *p* = 0.002], and ‘Reduce’ to ‘Increase’ [Wald *X*^2^ (1) = 15.5, *p* < 0.001]. Examining the assumption that the cab was green, pairwise comparisons were run to compare ‘No Change’ to ‘Increase’ [Wald *X*^2^ (1) = 2.4, *p* = 0.13], ‘No Change’ to ‘Reduce’ [Wald *X*^2^ (1) = 10.0, *p* = 0.002] and ‘Reduce’ and to ‘Increase’ [Wald *X*^2^ (1) = 8.8, *p* = 0.003].

Moving to the final row, where individuals were asked to update their estimate of the witness’s accuracy again after being told that subsequent investigations had found the cab really was green, only seven individuals increased their estimate of the witness’s accuracy. All others either reduced or made no change to their estimate. A binary logistic regression was run to test the effect of ‘Response type’ on the proportion reducing their estimate [Wald *X*^2^ (2) = 7.7, *p* = 0.02]. Pairwise comparisons were run to compare ‘No Change’ to ‘Increase’ [Wald *X*^2^ (1) = 3.8, *p* = 0.05], ‘No Change’ to ‘Reduce’ [Wald *X*^2^ (1) = 5.5, *p* = 0.02] and ‘Reduce’ to ‘Increase’ [Wald *X*^2^ (1) = 0.22, *p* = 0.64].

### Qualitative Data

Participants were asked to explain their reasoning after providing their posterior change estimate for the Witness’s accuracy. These were coded blind to response type by the first author. Four major codes were identified, but around half of all responses were also coded as ‘Unclassified’ where an understanding of the participants’ response could not be confidently attained. The first author gave their codebook containing these five codes (Table 2) to the third author. The third author then assigned these codes, blind to both response type and to the first author’s assignments. Inter-rater agreement was 78.6%, with disagreements generally being whether a response should be ‘unclassified’ or not. For the discrepant responses (28 total), if one coder had chosen ‘unclassified’ we assigned this code in order to be conservative – 22/28 of these were therefore classified that way. The remaining six were resolved through discussion. The proportion of each response type assigned each code post-agreement can be seen below in Table 2. Among responses that could be classified, one modal code stands out for each, however, for ‘No Change,’ a substantial amount were also coded as ‘Witness probably correct,’ similar to the ‘Increase’ responders. These will be discussed below.

**TABLE 2:** Percentages of each response type assigned each code type (modal code excluding ‘unclassified’ is highlighted for each response).

	Witness probably correct	Irrelevant	Witness probably Incorrect	Requires Certainty	Unclassified
Increase	33.3	3.3	6.7	–	56.7
No change	17.8	32.9	8.2	2.7	38.4
Reduce	3.6	–	50.0	–	46.4

#### Increase

The modal code assigned among ‘Increase’ responders was ‘Witness probably correct.’ This was assigned where the participant indicated that they thought the witness was likely to be correct or showed confidence in the witness. A selection of these responses can be seen in Table 3.

**TABLE 3:** A selection of ‘Increase’ responders open-text explanations of their reasoning assigned the code ‘Witness probably correct’.

*“Because he got another car right so it is 5/6.”*
*“… Now with 80% accuracy of the witness, stating that it is from the blue one, I am sure that the cab is from the blue company.”*
*“The witness has said they saw a blue cab.”*
*“I believe the witness would have seen correctly even under pressure.”*
*“They did do the trial 5 times and out of the 5 times they got it correct 4 times, had this been lower then I would have questioned the accuracy but 4 out of 5 is quite good.”*

#### No Change

The modal code among ‘No Change’ responders (30.8%) was ‘Irrelevant.’ This was assigned where the participant stated that the report by the witness has no bearing on their accuracy level. A selection of these responses can be seen in Table 4.

**TABLE 4:** A selection of ‘No Change’ responders open text explanations of their reasoning assigned the code ‘Irrelevant’.

*“Still 90% because the facts are still the same.”*
*“I don’t think the probability of what they saw regarding colors will affect the accuracy of their statement.”*
*“The report doesn’t change their estimated accuracy.”*
*“For me nothing changed because we have no new viable information.”*
*“Because the probability of it being a blue car and then the witness identifying it as a blue car are separate, so even if it’s a low probability, it wouldn’t affect their perception unless they were told beforehand that [it was] low probability.”*
*“The result of the test (blue or green) doesn’t change the level of accuracy of the witness.”*
*“They still got 4/5 trials right, so I’m still confident in them.”*

When told at the end of the experiment that the cab really was green, and the witness was incorrect, we can see that half of ‘No change’ participants still made no change to their estimate of the witness’s accuracy. A selection of these participants’ explanations of those responses can be seen in Table 5.

**TABLE 5:** A selection of ‘No Change’ responders open text explanations of their reasoning after being told the witness was incorrect and still making no change to their estimate of the witness’s accuracy.

*“The witness managed to get the correct color 4/5 based on the test. 1/5 times the witness fails and this was one of the situations where they failed.”*
*“I don’t feel that I can judge their accuracy based on this as this result could have been in the 20%”*
*“No remains 80%. The 20% percent would be them getting the color wrong.”*
*“I believe their accuracy is still not in question, they still had a 1 in 5 chance to get it wrong.”*
*“The previous test measured that the witness had a 4/5 chance to get the color correct. The accuracy still stands.”*
*“It fits 20% of not getting the right color.”*
*“There was still 20% chance he was wrong.”*
*“He has 4/5 so the car could be the 1/5.”*

#### Reduce

The most prominent code among ‘Reduce’ responders was ‘Witness probably incorrect.’ This was assigned when the participants stated that the witness was probably incorrect on this occasion, or expressed low confidence in them. Some of these also referenced the low base rate for blue cabs. A selection of these responses can be seen in Table 6.

**TABLE 6:** A selection of ‘Reduce’ responders open text explanations of their reasoning assigned the code ‘Witness probably incorrect.’

*“Based on the potential cab colors, it’s more likely than not that the cab was green, so I’m slightly more inclined to doubt the witness.”*
*“Very unlikely that it was a blue cab, since only one out of 10 plates were blue.”*
*“Because people can think they saw a thing and can be another completely different.”*
*“From a statistical point of view it is likely that the witness was wrong.”*
*“It was considerably less likely to be blue than green; this coupled with the one incorrect trial result, makes me less confident that the witness is correct. However, they still actually could be.”*
*“If was dark, how he/she can know whether car blue or green?”*
*“I think the accuracy of the witness became less than 80% because the probability that was a green cab is higher than her accuracy.”*
*“It’s hard to identify the color of a moving car at night, besides blue and green at high speed are easy to mistook for each other.”*

## Discussion

In this paper we aimed to examine responses to a modified version of the classic taxi cab problem including second order uncertainty. Through mixed methods, we aimed to uncover participants’ approaches to handling the new dynamics introduced in the modified version. Of principal interest was how participants altered their estimate of the witness’s accuracy after the witness reported that the cab was blue. We found that around half made no change, with around a quarter each reducing/increasing their estimate of the witness’ accuracy. Through convergent evidence, combining quantitative and qualitative responses we present below a general picture of the cognitive processes involved in each response type, however, we do not assume each represents a single coherent population (multiple cognitive processes may lead to the same response) and in some cases suggest this might be the case.

### Increase

‘Increase’ responders appear to be the most homogeneous of the three response types, with 80% self-reporting as having assumed the witness was correct, and, outside of the large proportion ‘unclassified,’ the majority of their open text data being coded as ‘Witness probably correct.’ Interestingly, none of these responders explicitly say they are ignoring the base rate data, or that they trust the witness more than the base rate data. They generally just only refer to the witness data in their responses, expressing confidence in them or belief they are correct, and typically not mentioning the base rate data at all. These responders cognitive process may therefore represent a form of base rate neglect (e.g., Tversky and Kahneman, 1974; Bar-Hillel, 1980). However, as can be seen in Figure 6 and Table 1, these participants certainly saw the base rate data as relevant before the witness made their report, suggesting a simple disregard for the relevance of that information is not a good explanation. However, Bar-Hillel (1980) proposed a ‘dominance’ theory of base rate neglect in such problems, where the piece of information seen as least relevant would be entirely disregarded, presumably for reasons of computational simplification. It is possible that our participants, once the witness report is provided, find the prospect of integrating these two figures too daunting. From there, finding the witness report more compelling than the base rate data for whatever reason, they may disregard the base rate, leading to an 80% estimate that the cab is blue based solely on the witness’s accuracy. However, this cannot provide a full explanation of the present results. Even if these participants do believe there is an 80% chance that the cab is blue, how does this justify increasing their estimate of the witness’s accuracy?

A similar response was also detected in other papers by the authors on reasoning with propensities (Dewitt et al., 2018,2020). In the scenario presented in both those papers, two nations are testing their missile detonation capabilities. Nation *X* has so far had only 1 success out of 6 attempts while *Y* has had 4 successes out of 6. Another missile then successfully detonates on the border between the two nations but we’re unable to detect the source. The key question, instead of who launched this missile (equivalent to whether the cab is blue), is what the new proficiency estimate for each nation is (equivalent to updating the witness’s accuracy). In both papers we found a similar approach to the present paper, where, when updating proficiencies, 1/3 of participants increased their estimate of *Y*’s proficiency, making no change to *X*. Because *X* and *Y* represent exhaustive and exclusive causes of the latest explosion, unlike in the present scenario, we were able to infer that they have treated *Y* launching the latest missile as a certainty (i.e., they have ‘given’ the whole responsibility for the detonation to *Y*, and none to *X*). In Dewitt et al. (2018) we also saw similar open text reasoning, with the majority of these participants simply stating that they believed *Y* was probably responsible. We labeled these participants as ‘categorical’ responders as we found that while they rated the probability that *Y* was responsible as 77.7% on average, they all treated it as a 100% certainty when updating their estimate. This treatment of a probabilistic variable (e.g., 80% estimate that the cab is blue based on the witness’s report) as a certainty may also be occurring in the present experiment. Evidence for this comes from the vast majority of ‘Increase’ responders who self-report as having ‘assumed’ the witness was correct.

Importantly, in both scenarios, this response has a circularity to its logic. In the missiles experiment, *Y* is assumed to have launched the missile based upon their previous success with missiles. Their success with missiles is then updated based upon the assumption that *Y* launched the missile (which, to reiterate the circularity, is based upon their past success with missiles). Similarly, in the present scenario, the only evidence that participants have that the witness might be correct this time is their previous accuracy. But just like in the missiles scenario, this is the very thing we want them to update. So, when this approach is adopted, it appears that once a witness gets to a certain level of trust, they will not only be assumed to be correct based only on this historical accuracy, but also, based on that assumption, they will be seen as even more accurate afterward, even when the only other evidence available actually strongly suggests they are incorrect. This has the same circularity as has been observed in confirmation bias and belief polarization literature (Lord et al., 1979; Plous, 1991; Nickerson, 1998; Cook, 2016; Fryer et al., 2019).

The treatment of probabilistic variables as categorical variables has also been previously reported under the names of ‘as if’ reasoning (Gettys et al., 1973) and ‘digitization’ (Johnson et al., 2018). Both sets of authors have found that in multi-step reasoning, where the output of one probabilistic calculation is used in a second calculation, the first output is often digitized (or, turned into categorical form) for the second calculation. For example, if one has to calculate the chance of rain, and then use that probability to estimate the chance that a party will be canceled, they will treat the chance of rain in that second calculation as either 0 or 1. In our missile launching scenario, this ‘multi step’ explanation for categorization was considered plausible, as the categorical response involved multiple steps (one first had to normalize a 66.6:16.6 ratio to get to the 80:20 probability of who launched the missile before using this latter value to update propensities). However, the present problem does not involve multiple steps as the participants are directly provided with the probabilities of, e.g., the cab being green and the witness being correct. While these are admittedly estimated from frequencies (9/10 and 4/5) this is not a true ‘first’ calculation in the sense meant by those authors (e.g., if participants had to first multiply two figures together to get the witness’s accuracy). Therefore, despite this problem not fitting the ‘multi-step’ format, we still saw large numbers of participants taking what appears to be a similar categorical approach. This may suggest that this phenomenon of digitization or categorization is a more general strategy to simplify a difficult problem (with multiple steps being just one source of difficulty). The current scenario unfortunately presents a situation (with two diagnostically opposite pieces of data) where such a strategy is at its most inappropriate (unlike, e.g., if one of the figures was close to 50:50), and so it is interesting that we still see such a strategy employed. It may be valuable to determine in future work if individuals are sensitive to the ‘appropriateness’ of this strategy when choosing to employ it, by varying the figures in the problem. It should also be noted that, even if participants are aware that it is not an ideal approach to the scenario, they may feel that they lack an alternative approach. Indeed, in Dewitt et al. (2020) we found that 1/3 of categorical responders endorsed the statement ‘I approximated that *Y* was entirely responsible for the launch in order to make the problem simpler but know this is not strictly accurate,’ suggesting some awareness that their approach was not fully normative.

### Reduce

‘Reduce’ responders appear to be more mixed than ‘Increase’ responders in their choices on the ‘assumption’ question. Around half self-report as assuming the cab is green, but around a quarter actually report they assumed the witness was correct, and another quarter report ‘Neither/other.’ However, almost all of those whose open text responses could be classified were coded as ‘Witness probably incorrect.’ Unlike ‘Increase’ responders, many of these did cite the base rate as a reason for this belief, saying either that the cab was very unlikely to be blue, or very likely to be green. Others stated low confidence or disbelief in the witness’s ability to make the judgment.

We think it is possible that there are two sub response types here. First, would be the mirror image of the ‘Increase’ responders, who may be committing ‘base rate conservatism,’ neglecting the relevance or value of the witness’s claim, and entirely focusing on the base rate and treating that as a certainty. This would correspond to those 46.4% who self-reported as assuming the cab was green.

Second would be those who dealt with the problem normatively/probabilistically, integrating both variables together (even if not fully mathematically). As the base rate is stronger than the witness’s report (90% vs. 80%), this leads to the conclusion that the witness is more likely to be incorrect than correct. There is no equivalent process to this that would lead to ‘increase,’ which is another reason to suppose a single cognitive process for that response. This process may correspond to those 28.6% who selected ‘Neither/other’ (i.e., didn’t ‘assume’ either way). These participants may have, rather than neglecting one piece of information or the other (either the base rate or the witness information), have integrated both, concluding that the witness is more likely to be incorrect but still maintaining a probabilistic representation of the problem, rather than collapsing into assumption-based thinking.

### No Change

Participants who made no change are also less obviously homogenous than the ‘Increase’ responders. Interestingly, quite a large proportion state that they assumed the witness was correct, despite not increasing their estimate of the witness’s accuracy. Similar to ‘Reduce’ responders, around a quarter self-reported assuming ‘Neither/other.’

While the main qualitative code for ‘No change’ responders was ‘Irrelevant,’ and only a few were coded as ‘Requires uncertainty,’ it is still very possible that uncertainty is a major reason for this response. These participants tended to be quite unforthcoming in their reason for why they think the new information is irrelevant, simply stating that they don’t see the connection. The reason for the new information being irrelevant could therefore very well be that we don’t yet know whether the cab really is green or blue – they may consider uncertain information irrelevant for updating the witness’s accuracy. This would fit with the ‘missiles’ experiments mentioned above (Dewitt et al., 2018,2020) where ‘No change’ was also a dominant response (about 1/3 both samples). In Dewitt et al. (2020), when asked to pick from a set of statements as to which most closely matched their reasoning, around two thirds of these responders chose ‘The evidence states it’s uncertain who launched the successful missile so you cannot change the proficiencies based on uncertainty.’

Another reason to think that uncertainty may be an important underlying reason for the ‘No change’ response is that half of these participants, when told that the cab really was green at the end of the experiment (and therefore that the witness was incorrect), reduced their estimate of the witness’s accuracy. This suggests that they do see the connection between the witness’s report that the cab was blue and their accuracy, but only once they know for certain that that report really is wrong. Indeed, while under uncertainty, participants may prefer to err on the side of avoiding updating incorrectly (Anderson, 2003).

However, a much larger percentage of ‘No change’ responders than either ‘Reduce’ or ‘Increase’ continue to make no change even when told that the witness was incorrect. A selection of responses which may indicate these participants’ thought processes can be seen in Table 5. There is a strong theme here of these participants seeing the latest failure of the witness as ‘fitting within’ the original accuracy estimate of 80%. In some cases, they seem to suggest that this could be ‘the one’ they got wrong (out of 5), which seems obviously incorrect given that previous information told them they already got one wrong during the tests. It is difficult to tell therefore whether this represents a simple misunderstanding of that, or whether there is a deeper and more interesting process occurring. Indeed, another interpretation is that these participants see one additional data point (even if that data point is now certain) as not enough to change a propensity based on five data points, when that propensity allows for some failure (i.e., the 20%). These participants may be seeing this latest claim as the first data point in another ‘run’ of 5, and while this first one failed, the next four may be successes, matching the original ‘80%.’ If true, there therefore seems here to be an over-sanctification of the original run of data, and furthermore, a similar tendency to ‘wait for more data before updating’ as with the single no change response.

## Conclusion

Overall, these findings seem to represent a general unwillingness or inability by at least 3/4 of our participants to deal with the problem probabilistically when answering second order questions, either converting those variables into categorical form (those ‘Increase’ and ‘Reduce’ responders who ‘assumed’ either way), or withholding judgment until they are certain (‘No change’ responders). This appears to represent a major departure from a Bayesian treatment of the problem, where any information about the state of one variable (i.e., the witness’s report), even if probabilistic, can be used to update our estimates for other causally related variables (i.e., the witness’s accuracy).

Generally therefore, in studying responses to this modified taxi cab problem, we have corroborated findings in previous work. The present work and the missiles work probe participants reasoning in different ways, and the problems have slightly different dynamics, and yet both point toward a substantial majority of participants adopting a categorical representation when updating propensities. Two approaches seem to stem from this. Some participants refuse to update entirely, until the state of the event is known. Other participants seem to convert the event into a certainty one way or the other, and update propensities based upon that assumption. While the issue with the former approach is to make no use of valuable information, the latter may be more damaging. We have already spoken about the circularity of the ‘assume witness correct’ approach. Before even knowing whether the witness is correct, the mere fact that the witness has shown themselves to be fairly reliable in the past, seems to lead these individuals to increase their trust in them following their claim, as if they knew the witness was correct this time. This suggests that once a person or system reaches a certain level of trust, they may be able to make claims, and even without the truth of these being determined, are not only trusted in the individual situation (which may be reasonable), but even have trust in them increased for the future on the assumption they probably were correct this time. This is perhaps all the more troubling given that the previous accuracy estimate in this experiment was based upon a very small number of tests of the witness. With such a small number of trials, it is highly possible that the witness just got lucky on a couple of occasions. With only two options to guess from, it is quite possible that they are at chance level for judging cab colors under the given conditions. Therefore, in combination, this seems to suggest that if an individual gets lucky with a few accurate claims early on, and they pass the ‘safe to assume they are correct’ threshold, their early luck can take on a self-reinforcing dynamic, where trust in them is further enhanced even without further verification of their claims. This dynamic was also discussed in Dewitt et al. (2020) in the context of prejudice toward an individual or group of producing some negative outcome. Indeed, it may be that this ‘assumption’ approach lends any situation where we are estimating a propensity (whether for a ‘good’ or ‘bad’ outcome) a positive feedback dynamic similar to that seen in confirmation bias/belief polarization literature.

## Data Availability Statement

The original contributions presented in the study are publicly available. This data can be found here: https://osf.io/q68cu/.

## Ethics Statement

The studies involving human participants were reviewed and approved by UCL Department of Experimental Psychology. The patients/participants provided their written informed consent to participate in this study.

## Author Contributions

This work builds upon previous work by the current authors. SD conceived of applying those previous ideas to the present problem, developed, ran and analyzed the experiment, and was the primary author. Ideas were discussed and refined with DL throughout the entire process, including the analysis of results and DL provided thorough feedback on various drafts of the manuscript. NF provided the causal Bayesian network model and technical advice throughout including feedback on the manuscript. AL provided second coding for the qualitative data and feedback on the manuscript. All authors contributed to the article and approved the submitted version.

## Conflict of Interest

The authors declare that the research was conducted in the absence of any commercial or financial relationships that could be construed as a potential conflict of interest.
